# 3D Artificial Skin Model As a Novel Strategy for the Detection of Pyroptosis-Cascade Activation in Amyotrophic Lateral Sclerosis

**DOI:** 10.1021/acsami.5c23366

**Published:** 2026-03-06

**Authors:** Enrico Scarpa, Ugo D’Amora, Noemi De Cesare, Irene Bonadies, Raffaele Dubbioso, Maria Nolano, Principia Dardano, Luca De Stefano, Alessandra Fasolino, Stefania Zeppetelli, Alessandro Silvestri, Chiara Zanardi, Evelina Milella, Ines Fasolino

**Affiliations:** † 119618 Institute of Polymers, Composites and Biomaterials − National Research Council (IPCB-CNR), Viale Kennedy 54, Mostra d’Oltremare pad. 20, 80125 Naples, Italy; ‡ Institute of Polymers, Composites and Biomaterials − National Research Council (IPCB-CNR), Via Campi Flegrei 34, 80078 Pozzuoli, Italy; § Department of Neurosciences, Reproductive Sciences and Odontostomatology, 9307 University of Naples Federico II, Via Sergio Pansini, 5, 80131 Naples, Italy; ∥ Laboratorio Biopsie di Cute, Istituti Clinici Scientifici Maugeri Spa SB − IRCCS Telese Terme, Via Bagni Vecchi, 1, 82037 Telese Terme (BN), Italy; ⊥ Institute of Applied Sciences and Intelligent Systems − National Research Council (ISASI-CNR), Via Pietro Castellino, 111, 80131 Naples, Italy; # Clinical Neurophysiology Unit “A. Cardarelli Hospital”, 9, 80131 Naples, Italy; ○ Department of Molecular Sciences and Nanosystems, Ca’Foscari University of Venice, via Torino 155, 30172 Venezia, Italy

**Keywords:** pyroptosis, nerve degeneration, 3D printed artificial skin, ALS, diagnosis

## Abstract

Amyotrophic lateral sclerosis (ALS) is a severe adult-onset neurodegenerative disease with limited treatment approaches. Evidence has shown that degeneration of cutaneous nerves may reflect neurodegenerative processes occurring within the central nervous system. Although skin biopsy is widely adopted in clinical practice, the procedure is invasive and requires multiple patients’ tissue removals. Therefore, we developed a 3D innervated skin model by combining 3D printing of methacrylated hyaluronic acid as an innovative tool for better reproducing the dermis and epidermis and electrospinning of polylactic acid for mimicking skin innervation. Later, 3D artificial skin was colonized with a preneuronal cell line (SH-SY5Y) and fibroblasts isolated from skin biopsy of ALS patients at different disease stages. 3D skin possesses a porosity suitable for cell colonization and a high stability. Importantly, biological results reveal an increase of TAR DNA-binding protein 43 aggregates, NOD-like receptor pyrin domain containing protein 3, interleukin (IL)-18, IL-6, and nitrites in 3D skin of ALS patients, thus indicating pyroptosis activation linked to neurodegeneration. This physiologically relevant 3D skin model reduces the need for repeated biopsies, allows standardized experimental conditions, and supports biomarker research and preclinical drug testing in ALS.

## Graphical abstract

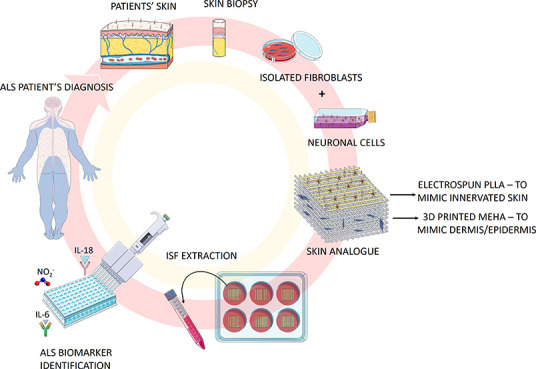

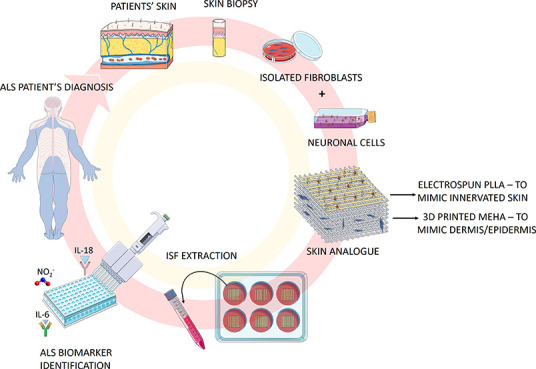

## 1 Introduction

Amyotrophic lateral sclerosis (ALS) is a lethal neurodegenerative disease characterized by the progressive degeneration of motor neurons in both the brain and spinal cord, leading to symptoms ranging from muscle weakness to respiratory failure.^1^ ALS has no defined etiology and occurs in both sporadic and familial forms. Genetic and environmental factors contribute to the disease risk. Despite these advances, the disease still remains incurable. Treatment focuses on symptom management, use of riluzole, and support for patients and caregivers.^2^ The lack of validated biomarkers limits early diagnosis, prognostic stratification, and development of targeted therapies. This gap contributes to diagnostic delay, which often approaches one year from symptom onset, and reduces the window for timely intervention. Median survival remains about three to five years from symptom onset.^3^ Although ALS primarily affects motor neurons, sensory and autonomic involvement also occurs. Studies of skin punch biopsies show degeneration of cutaneous innervation, with reduced intraepidermal nerve fiber density reported in about 80% of patients. These findings support the concept of systemic neurodegeneration beyond the motor system.^4–6^ However, routine use of skin biopsy in clinical and diagnostic settings remains challenging, especially in early disease stages.^7^ The procedure is invasive, requires specialized expertise, and often needs repeated sampling.^8^ Identifying reliable, minimally invasive biomarkers remains a priority to improve early detection, support patient stratification, and monitor disease progression in clinical trials.^7^ Therefore, different approaches must be taken into account. Recently, the use of synthetic human skin equivalents as *in vitro* 3D models has increased due to advancements in materials science and engineering.^8^ The use of 3D artificial skin offers the great advantage of reducing the number of biopsies performed on patients, thus overcoming drawbacks related to patient’ compliance. Furthermore, 3D artificial skin allows working in more standardized conditions compared to skin biopsies, thus obtaining samples (isolated cell lines) at a higher purity level. These skin models provide a safe, low-cost, and rapid substitute for animal experiments while also serving as a translational research platform thanks to their human origin. Because of their capacity to reproduce specific cellular ecosystems, 3D *in vitro* models also allow an easier and more direct investigation of certain cell interactions and molecule activities to accurately replicate different pathologies.^9^ In addition, the realization of innervated models improves *in vitro* testing by providing a more realistic cutaneous environment that is useful to study neurodegenerative disorders. Such an innervated skin model allows to study, from a molecular point of view, neurodegeneration and neuroinflammation mechanisms underlying pathological states. While the exact cause of ALS is still unclear, increasing research shows that neuroinflammatory processes including microglial activation, astrogliosis, and infiltration of immune cells represent a significant pathological hallmark in both the spinal cord and peripheral nerves of ALS patients and mouse models.^10^ It has been noted that the primary effector cells of neuroinflammation mediate both neuroprotective and neurotoxic effects through context- and time-dependent interactions. Characterizing the major contributors to this immune dysfunction and their role in the development of the illness is crucial, since ALS involves both systemic and local immune system changes. Inflammasomes, which are cytosolic protein complexes that function as intracellular sensors to detect external signals (PAMPs, pathogen-associated molecular patterns) and internal danger signals (DAMPs, damage-associated molecular patterns), have been found to mediate neuroinflammation in recent years. The best characterized inflammasome is NLRP-3, consisting of the NOD-like receptor pyrin domain containing protein 3, the adaptor protein apoptosis-associated speck-like protein containing a CARD (ASC) and pro-caspase 1 that causes inflammatory cell death (pyroptosis). Although it has been demonstrated that the NLRP-3 inflammasome activation plays a crucial role in neurodegenerative diseases, such as Alzheimer’s and Parkinson’s disease, current knowledge remains limited about its implication in ALS.^11^ For ALS, the research is mainly focused on inflammasome and pyroptotic cell death and carried out in preclinical models as well as in human *post-mortem* tissues. Currently, several studies have shown the activation of NLRP-3 inflammasome in the brains and spinal cord of SOD1-G93A mice models [transgenic mice express a G93A mutant form of human superoxide dismutase 1 (SOD1)] and ALS patients.^12,13^ In addition, in the brain of SOD1 transgenic model, the increase of NLRP-3 protein was related to the presence of active caspase-1, resulting in an increase in interleukin 18 (IL-18) and IL-1β levels, that in turn caused a higher expression of lymphocyte T-CD4, CD8, CD44, and CD68.^13^ Microglial NLRP-3 upregulation was also observed in the TDP-43Q331 K ALS mouse model.^14^ Furthermore, p-TDP-43 aggregates in the peripheral nervous system of ALS patients may constitute an accessible tissue biomarker alongside NLRP-3.^15,16^ Analogously, abnormal TDP-43 expression has been demonstrated in human peripheral nerves through nerve and skin biopsies.^17^ It was previously reported a reduction in distal intraepidermal nerve fiber density inALS patients with spinal-onset.^18^ Morphological alterations in cutaneous sensory and autonomic nerves were linked to a marked decrease in the dermal vascular network. Notably, a previously undescribed association between autonomic dysfunction and vascular impairment was identified, correlating with the rate of disease progression.^19^ Previously, we found that the correlation between vascular abnormalities and nerve degeneration suggests that inflammatory mechanisms may play a potential role in peripheral nerve degeneration.^19^ Specifically, it is worth noting that the overall data, collected until now, support the idea of a possible link between pathological TDP-43 accumulation, activation of microglia, pyroptosis cascade, and neurodegeneration.

Recently, our research group has already developed a 3D skin tissue using a chemically modified hyaluronic acid (HA) by bioprinting technique.^20,21^ High water content, softness, flexibility, and biocompatibility have boosted the use of hydrogels for recreating the *skin milieu*.^22^ Among them, HA has drawn special attention because it resembles the natural extracellular matrix (ECM) of a number of connective tissues and because it plays a role in biological processes related to tissue repair, such as inflammation, angiogenesis, and ECM organization.^23^ Our results suggested that the use of a bioprinted HA derivative, methacrylated HA (MEHA), allowed obtaining stable polymer networks.^20,21^ However, to the best of our knowledge, the use of a scaffold/patch as a 3D model for the diagnosis of ALS has never been exploited before.

On the other hand, electrospun poly-l-lactic acid (PLLA) microfibers have been widely used to investigate neuronal cell behavior in terms of biocompatibility and differentiation processes. To better understand the mechanisms behind neurodegenerative disorders, PLLA has been extensively studied in the presence of SH-SY5Y preneuronal cells. Furthermore, the incorporation of bioactive signals into PLLA microfibers greatly enhanced cell adhesion and differentiation, making them ideal candidates for neural tissue engineering.^24,25^ Furthermore, SH-SY5Y cells, when cocultured with skin cell lines, demonstrated to be functional to reproduce *in vitro* sensitive skin.^26^ Indeed, the SH-SY5Y human neuroblastoma cell line, typically employed as a model for adrenergic or dopaminergic neurons, also displays characteristics of peripheral sensory neurons, including the functional presence of sensory neuron-specific sodium channels.^26^ Prompted by those positive results, biofabrication and electrospinning technologies were coupled to manufacture a 3D complex model of innervated skin containing artificial interstitial fluid (ISF) as an efficient source of neuroinflammatory and neurodegenerative biomarkers. This 3D innervated artificial skin model was made up of a 3D printed MEHA region, for better reproducing the dermis/epidermis, and an electrospun PLLA area for mimicking skin innervation. Later, it was colonized with SH-SY5Y and fibroblasts isolated from the skin biopsy of ALS patients at different disease stages (Slow and Fast). According to previous studies, patient skin fibroblasts may be used as model systems for neurodegenerative diseases because skin fibroblasts present a system with defined mutations and the cumulative cellular damage of the patients.^27^ Finally, the immunodegenerative patient’s profile in terms of pyroptosis (inflammasome cascade) marker expression was defined through marker quantification on 3D skin tissue and ISF.

Herein, the present study introduces two major innovative aspects: (i) development of a 3D skin tissue model mimicking ALS patients’ skin and (ii) exploration of the link between ALS neurodegeneration and inflammasome cascade activation (pyroptosis). In this context, the first novelty lies in the development and validation of a 3D skin model designed to overcome the limitations associated with traditional skin biopsy collection and management. This approach minimizes ethical and logistical constraints while providing a physiologically relevant platform for the experimental studies. By offering a controlled and reproducible system, the model enables the investigation of neurodegenerative-related alterations without resorting to invasive procedures. The second innovative aspect focuses on elucidating the correlation between neuronal degeneration in ALS and the activation of the inflammasome pathway, particularly pyroptosis. This strategy not only deepens the understanding of immune-inflammatory mechanisms underlying ALS but also aims to identify novel biomarkers for early diagnosis and patient stratification. This perspective is groundbreaking because it shifts the focus from purely neuronal analysis to a systemic interaction using the skin as a window into neuroinflammation.

## 2 Materials and Methods

### 2.1 Collection of Skin Biopsies, Fibroblast Isolation, and *In Vitro* Cell Analysis

Skin biopsies were obtained from two patients with ALS at different disease stages according to the King’s College staging system and from healthy controls. These two patients, specifically, Patient 3 and Patient 6, belong to a cohort of six patients (Table S1) and underwent skin biopsy procedures for the standardization of the 3D skin model. The first patient was classified as Stage 2, with involvement of a second anatomical region and a rate of progression of 0.13. This patient was defined as an early stage and slow progressor (S-ALS). The second patient was classified as Stage 4A and 4B, with nutritional and respiratory failure requiring both feeding tube and noninvasive ventilation. The rate of progression was 1.64, consistent with a late stage fast progressor (F-ALS) profile (Table S1). All patients were carefully screened for potential confounders affecting the systemic inflammation. None had active neoplastic, infectious, autoimmune, or rheumatologic diseases. In addition, patients with diabetes, chronic inflammatory disorders, recent surgery or trauma, chronic use of immunosuppressive or anti-inflammatory medications, and acute intercurrent illnesses at the time of sampling were excluded. Patients were recruited at the ALS center of Federico II University. Written informed consent was obtained from all subjects according to the Declaration of Helsinki before enrollment in the study. The study protocol was approved by the local Ethics Committee (protocol numbers 100/17/ES01 and 151/2023). Skin biopsies by means of a 3 mm punch were obtained at baseline (T0) from the thigh on the more affected side in each ALS patient. Afterward, they were processed to quantify the expression of pyroptosis cascade markers and were compared with healthy controls represented by immortalized adult human dermal fibroblasts (HDF) purchased from Sigma-Aldrich, Milan, Italy.

Commercially available fibroblasts were used to avoid the possibility that fibroblasts isolated from “healthy” patients might introduce false positives when compared to fibroblasts obtained from ALS patients who are definitively affected by the disease. Indeed, commercial fibroblast lines are well-characterized, standardized, and free from subclinical or undiagnosed conditions that could influence the experimental outcomes. By relying on these validated cell lines, we ensured a more reliable baseline control and reduced the risk that hidden pathological features in patient-derived “healthy” fibroblasts could bias the comparison with diseased samples. Dermal fibroblasts were isolated from skin biopsies of ALS Slow and Fast progressors by using the protocol described by Iannello at al.^28^ This method does not require the use of enzymatic digestion or mechanical dissociation. Specifically, the biopsy was fixed on the surface of a tissue culture-plate treated with gelatin (Sigma-Aldrich, Milan, Italy), thus allowing the fibroblasts growth. Later, fibroblasts were cultured and processed with viability assay and immunofluorescence protocols to analyze growth, fibroblast marker, and morphological differences between the fibroblasts derived from Slow and Fast progressors and controls (HDF). Furthermore, SH-SY5Y cell two-dimensional (2D) characterization was performed before seeding cells on the 3D skin model for mimicking skin innervation. For this purpose, SH-SY5Y cells (2.0 × 10^4^ cells/well) were seeded in a 6-multiwell plate and grown in an appropriate proliferation medium. After 24 h, cell culture medium was removed and replaced with Dulbecco’s Modified Eagle Medium-F12 (DMEM-F12, Pan Biotech) containing 1% fetal bovine serum (FBS, Sigma-Aldrich, Milan, Italy) and 10 μM retinoic acid (RA, Sigma-Aldrich, Milan, Italy) for 7 days. After this time, for differentiation marker expression, cells were washed with phosphate buffered saline (PBS, Sigma-Aldrich, Milan, Italy) 1×, fixed with a solution of 4% w/v paraformaldehyde (PFA, Sigma-Aldrich, Milan, Italy) for 2 h at room temperature (RT) and permeabilized in 0.1% v/v% bovine serum albumin (BSA, Sigma-Aldrich, Milan, Italy) + 0.03% v/v Triton-100× solution (Sigma-Aldrich, Milan, Italy) for 1 h. Each well was then filled with FITC-conjugated GAP-43 (1:100 dilution), a marker protein unique to neurons, and incubated for the entire night at 4 °C. The cytoskeleton was stained for 1 h using anti-Phalloidin-ATTO 594 (red fluorescence staining, Molecular Probes, Life Technologies) solution (1:200) following three PBS washes. Finally, nuclei were stained with 4′,6-diamidino-2-phenylindole, dihydrochloride (DAPI, 10 μg × mL^–1^, blue fluorescence staining, Molecular Probes, Thermo Fisher Scientific). Images acquisition was performed by fluorescence microscope at magnification 10× (JuLI Stage by NanoEntek).

Later, fibroblasts and differentiated SH-SY5Y were seeded in a 12-multiwell plate at a density of 1.5 × 10^4^ cells/well and cultured for 7 days. This time point was selected to ensure complete cellularization of the 3D skin model. For cell proliferation, the experiment was carried out using the Alamar Blue assay, based on the metabolic activity of living cells. To this end, DMEM without Phenol Red (HyClone, UK) containing 10% v/v Alamar Blue (Biorad, Italy) was added to the cells and incubated for 4 h at 37 °C and 5% CO_2_. Then, 100 μL of supernatant were transferred in a 96-well plate and a spectrophotometer (VICTOR X3, 156 PerkinElmer, Milan, Italy) was then used to measure the absorbance at wavelengths of 570 and 600 nm. Cell proliferation was expressed as a percentage of cell viability. For morphological analysis and fibroblast marker expression, cells were grown for 7 days at density of 1.5 × 10^4^ in 12-multiwell plates and fixed with a solution of 4% w/v PFA for 2 h at RT and permeabilized in 0.1% v/v% BSA + 0.03% v/v Triton-100× solution for 1 h. The cells were then incubated with Fluorescein isothiocyanate (FITC)-conjugated Vimentin (Proteintech, Manchester, UK) (1:100 dilution) and/or heat shock protein 47 (Hsp47) (Proteintech, Manchester, UK) (1:100 dilution) overnight at 4 °C. Later, cells were washed with PBS 1× and Phalloidin-ATTO 594 (1:200) was incubated for 1 h at RT. After this time, cells were washed thrice with PBS 1× and nuclei were counterstained with DAPI, 10 μg × mL^–1^. After three washes in PBS 1×, cells were observed under a fluorescence microscope (JuLI Stage by NanoEntek). Fluorescence intensity was quantified through image analysis performed with ImageJ software (version 1.44, running Java 1.6 in 64-bit mode). The fluorescence intensity was normalized to the number of cells per surface, calculated by subtracting the cell intensity from the background intensity and expressed as mean of fluorescence intensity. Finally, p-TDP-43 and NLRP-3 expression was quantified on fibroblasts by using Western blot analysis. To this end, cells (fibroblasts derived from Slow and Fast progressors and controls) were seeded and grown for 7 days at density of 5 × 10^5^ in 6-multiwell plates. Later, cells were lysed in a RIPA buffer (Thermo Fisher Scientific) solution and processed for Western blot analysis. Proteins (10 μg) were resolved on 4–20% polyacrylamide gel and electrophoretically transferred to a nitrocellulose membrane (Bio-Rad). Membranes were blocked in 5% nonfat skim milk (Sigma-Aldrich, Milan, Italy) for 1 h at RT and incubated with primary antibodies against NRLP-3 (1:500, Abcam, IT) or p-TDP-43 (1:500, Proteintech, Manchester, UK), overnight at 4 °C. Afterward, membranes were washed with PBS-Tween 0.1×, incubated with HRP-conjugated secondary antibodies at 1:1000 dilution (Biorad) for 1 h, and washed. The signals were visualized by an enhanced chemiluminescence kit (Clarity Western ECL Substrate, Bio-Rad) using a VersaDoc MP 5000 System (Bio-Rad Laboratories, Inc.) and analyzed with Quantity One Software version 4.6.3. Loading controls (GAPDH, 1:1000 dilution, Abcam) were used for protein expression normalization, and the results were expressed as arbitrary units.

### 2.2 3D Skin Model Design and Characterization

#### 2.2.1 3D Printing of Methacrylated Hyaluronic Acid and Electrospinning of Polylactic Acid

High molecular weight hyaluronic acid sodium salt (HA, *M*_w_ = 1.5–1.8 × 10^6^ Da from *Streptococcus equi*, Sigma-Aldrich, Milan, Italy) was metahcrylated to obtain MEHA according to previously published papers.^20,21^ Attenuated total reflection–Fourier transform infrared (ATR-FTIR) analysis was performed by a Thermoscienfic, Nicolet Summit X FTIR spectrometer (Waltham, Massachusetts, USA) to identify the functional groups of MEHA. Dried MEHA and HA (control) were scanned from 500 to 4000 cm^–1^ with a spectral resolution of 2 cm^–1^, 64 scans.

Freeze-dried MEHA (4% w/v) was dissolved in distilled water with 0.1% w/v 2-hydroxy-4′- (2-hydroxyethoxy)-2-methylpropiophenone (Irgacure 2959, Sigma-Aldrich, Milan, Italy). “Rokit Invivo 4D2” (Rokit Healthcare Inc., Seoul, Korea, 1.80 firmware) was used for 3D printing. New Creator K 1.57.70 was employed to slice the input printing model into a grid pattern. A speed of 6 mm × s^–1^ was used for printing. The bed was set to 0 °C, and the dispenser was set to 15 °C. Then, 20 mm × 20 mm × 3 mm porous structures were manufactured by using a 0.6 mm needle, a 0.4 mm layer thickness, and a 50% fill density. UV light (λ: 365 nm) was employed to cross-link the biomaterial ink during printing, improving its mechanical features and preventing the structures from collapsing. Following printing, 3D porous structures were also post-cross-linked for 10 min in a UV cabinet (Analytik Jena UVP cross-linker, CL-1000, λ: 365 nm). Prior to use, the structures were stored at −80 °C after being freeze-dried for 24 h using LaboGene’s CoolSafe 55 – 4 PRO, Bjarkesvej, Denmark. Electrospun PLLA (Ingeo 4032D, NatureWorks LLC) fibers were fabricated via an Electrospinning Setup NF103 MECC Co., Ltd. (Fukuoka, Japan) equipped with a single nozzle and a parallel electrode to obtain highly aligned meshes. To this aim, PLLA was solubilized in 10% w/v in chloroform/dimethylformamide 90/10 (Sigma-Aldrich, Milan, Italy), according to Fasolino et al.^29^ Process parameters were optimized and fixed at a flow rate of 3 mL × h^–1^, voltage of 25 kV, and nozzle-collector distance of 30 cm to obtain defect-free fibers. Electrospinning was conducted at RT and 10% relative humidity. Then three layers of electrospun meshes were stuck on a 3D printed structure, obtaining a complex 3D skin model named PLLA@MEHA. Fiber average diameter, fiber orientation and fiber density were calculated from scanning electron microscopy (SEM, FEI Quanta 200 FEG, Eindhoven, The Netherlands) images by using ImageJ1.48i software. Thirty fibers were used for diameter calculation, and three images at 4000× magnification were used to assess fiber orientation and density. The ImageJ plugin called directionality was utilized to determine the angle distribution with respect to the horizontal axis of the image; the angle distribution was divided from −90° to +90°. The ImageJ plugin called Cell Counter was employed to assess the fiber number within a fixed distance orthogonal to the fiber alignment direction (#/μm).

#### 2.2.2 Morphological, Physicochemical, and Mechanical Characterization of PLLA@MEHA Skin Model

Morphology of the PLLA@MEHA skin model was investigated by SEM. To such a purpose, an ion sputter was used to apply an ultrathin layer of gold/platinum (Au/Pt) onto lyophilized samples.

By analyzing the weight changes of the structures over time, gravimetric measurements were used to evaluate the swelling behavior of the PLLA@MEHA skin complex model. To replicate the *in vitro* cell culture conditions and timing of *in vitro* cell culture research, five freeze-dried structures were weighed (*w*_dry_) and immersed up to 6 h in DMEM (5 mL) completed with antibiotics at pH 7.4 and 37 °C. Therefore, after gently tapping on filter paper to remove the extra liquid, the hydrated structures were weighed (*w*_swollen_) at the current time points. Lastly, equation 1 was used to determine the mass swelling ratio (*Q*):

1 $$Q=\frac{w_{swollen}-w_{dry}}{w_{dry}}$$

For the stability studies, samples were collected, frozen at −80 °C, lyophilized, and weighed (*w*_t_) at predetermined intervals (1, 7, and 14 days). Equation 2 was used to determine the weight loss (%):

$$Weight\;Loss\;\left(\%\right)=\frac{w_{dry}-w_{t}}{w_{dry}}\times 100$$ 2

Uniaxial compression testing was performed on MEHA and PLLA@MEHA block-shaped specimens (20 mm × 20 mm × 3 mm) at a crosshead speed of 1 mm × min^–1^ until a strain of 0.4 mm × mm^–1^ (40%) was reached. Wet samples were placed on the lower plate and compressed by the upper plate attached to a load cell. The initial compressive modulus was measured from the average slope of the stress–strain curve within the 0–10% strain range. For each material, five to six specimens were tested using an INSTRON 5566 (Bucks, UK) universal testing machine at RT. Results are expressed as the main value ± standard error of measure (S.E.M.).

### 2.3 Biological and Biochemical Characterization of PLLA@MEHA Skin Model

#### 2.3.1 3D Skin Model Biocompatibility

3D artificial skin was colonized with differentiated SH-SY5Y and fibroblasts isolated from the skin biopsy of ALS patients at different disease stages (Slow and Fast). Specifically, for each 3D structure, SH-SY5Y cells (2 × 10^4^ cells, passage 15) were seeded on electrospun PLLA surface for mimicking skin innervation, meanwhile fibroblasts (2 × 10^4^ cells, passage 2) isolated from skin biopsy of ALS patients at different disease stages (Slow and Fast) or HDF cells as healthy control, were seeded on 3D printed MEHA for better reproducing dermis/epidermis. After 7 days of cell coculture, cell viability was assessed via Alamar Blue assay as detailed above.

For morphological analyses, at day 7 of cell culture, the samples were fixed with a solution of 4% w/v PFA overnight at 4 °C. After this time, samples were washed thrice with PBS 1× and permeabilized in 0.1% v/v BSA + 0.03% v/v Triton-100× solution for 1 h. Samples were then washed three times with PBS 1× and stained with FITC-conjugated phalloidin (Thermo Fisher Scientific, Waltham, Massachusetts, USA) solution for 1 h. Later, samples were washed, and nuclei were counterstained with 10 μg × mL^–1^ DAPI. The samples were washed with PBS 1× thrice and observed through fluorescence microscope at magnification 10× (JuLI Stage by NanoEntek).

Cell adhesion on 3D artificial skin was confirmed by using SEM. For this purpose, 2 × 10^4^ cells for each cell line were seeded onto the 3D structure and grown for 7 days. After this time, the cells were fixed on samples with a solution of 4% PFA overnight at 4 °C. The samples were washed and dehydrated through a graded ethanol series, then mounted onto aluminum stubs using double-sided adhesive tape and subjected to a 20 kV accelerating voltage. Subsequently, they were coated with a 10 nm Au layer via thermal evaporation at a deposition rate of 0.1 Å × s^–1^. The samples were morphologically characterized using field emission scanning electron microscopy (FESEM, Carl Zeiss Gemini 460). To maximize surface resolution and minimize beam-induced damage or charging artifacts, imaging was carried out using an accelerating voltage of 5 kV and a beam current of 85 pA. A secondary electron (SE) imaging detector was employed, which is particularly sensitive to surface topography and allows for detailed visualization of nanoscale features and surface texture.

#### 2.3.2 Pyroptosis and Neurodegenerative (NLRP-3 and p-TDP-43) Marker Expression

p-TDP-43 and NLRP-3 expressions were quantified also on 3D artificial skin through fluorescence analysis. To this end, fibroblasts derived from Slow and Fast progressors and controls (2 × 10^4^) were cocultured with SH-SY5Y cells (2 × 10^4^) onto MEHA and PLLA surfaces, respectively, for 7 days. Later, samples were fixed in 4% w/v PFA overnight at 4 °C and permeabilized in 0.1% v/v% BSA + 0.03% v/v Triton-100× solution for 1 h. The cells were then incubated with anti-p-TDP-43 or NLRP-3 (1:500 dilution) overnight at 4 °C followed by incubation with Rhodamine (TRITC)–conjugated or Alexa Fluor 488 goat antirabbit IgG antibodies (1:1000, Molecular Probes, Life Technologies) for 1 h. After three washes in PBS 1×, the cytoskeleton was stained with anti-Phalloidin-ATTO 594 or FITC (fluorescein 5(6)-isothiocyanate)-conjugated phalloidin (Thermo Fisher Scientific, Waltham, Massachusetts, USA) solutions (1:200) for 1 h. Finally, nuclei staining was performed through DAPI (10 μg × mL^–1^) incubation for 10 min at 37 °C. After three washes in PBS 1× cells were observed under a fluorescence microscope (JuLI Stage and Leica DMi8 microscopes).

#### 2.3.3 Nitrites and Pyroptosis Cytokine Levels

ISF was extracted from the 3D skin model through needle sampling. Later, ISF samples were centrifuged for 20 min at 10,000 rpm at 4 °C to remove any cellular contaminants and debris. For nitrite level production, 100 μL of ISF were incubated into a 96-multiwell plate with an equal volume of Griess reagent (Sigma-Aldrich, Milan, Italy) and the absorbance was measured at 550 nm after 1 h of incubation at RT using a fluorescent microplate reader (VICTOR X3, 156 PerkinElmer, Milan, Italy). IL-6 and IL-18 levels were quantified on ISF obtained from 3D artificial skin using commercial ELISA kits (Proteintech) according to the manufacturer’s instructions and compared with levels obtained in 2D culture conditions. Enzyme-linked immunosorbent assay (ELISA) results were measured as the optical density with a spectrophotometer (Victor X3, PerkinElmer, Milan, Italy) at wavelengths of 450 nm.

### 2.4 Statistical Analysis

GraphPad Prism, version 8.00 (GraphPad Software, La Jolla, California, USA), was used to conduct statistical analysis. When necessary, Sidak’s multiple comparisons test, two-way ANOVA, and the student’s *t* test were used. The findings are presented as mean ± standard deviation of mean (S.E.M.) or mean ± standard deviation (S.D.). Significant values were defined as *p* < 0.05.

## 3 Results and Discussions

### 3.1 Synthesis and Characterization of 3D Skin Model

3D printed PLLA@MEHA models were obtained by suitably combining 3D printing and electrospinning technologies (Figure 1A) to mimic the two compartments devoted to the innervated skin as well as the dermis/epidermis. MEHA and PLLA were chosen as best candidates based on our previous studies, respectively, on skin and neural tissue engineering.^20,21,29^ However, to the best of our knowledge, there are no papers focusing on the combination of those two polymers processed by additive manufacturing and electrospinning, respectively. Certainly, such complex constructs have never been employed as *in vitro* models to study the diagnosis and progression of ALS. Specifically, the combination of MEHA and PLLA using the two additive manufacturing techniques resulted in an ALS *in vitro* model of groundbreaking fidelity and realism. Thus, the development of composite skin models featuring both macro- and microporous architectures, achieved through the integration of 3D printed hydrogel scaffolds and electrospun microfibers, holds significant promise in the biomedical field.

**Figure 1:**
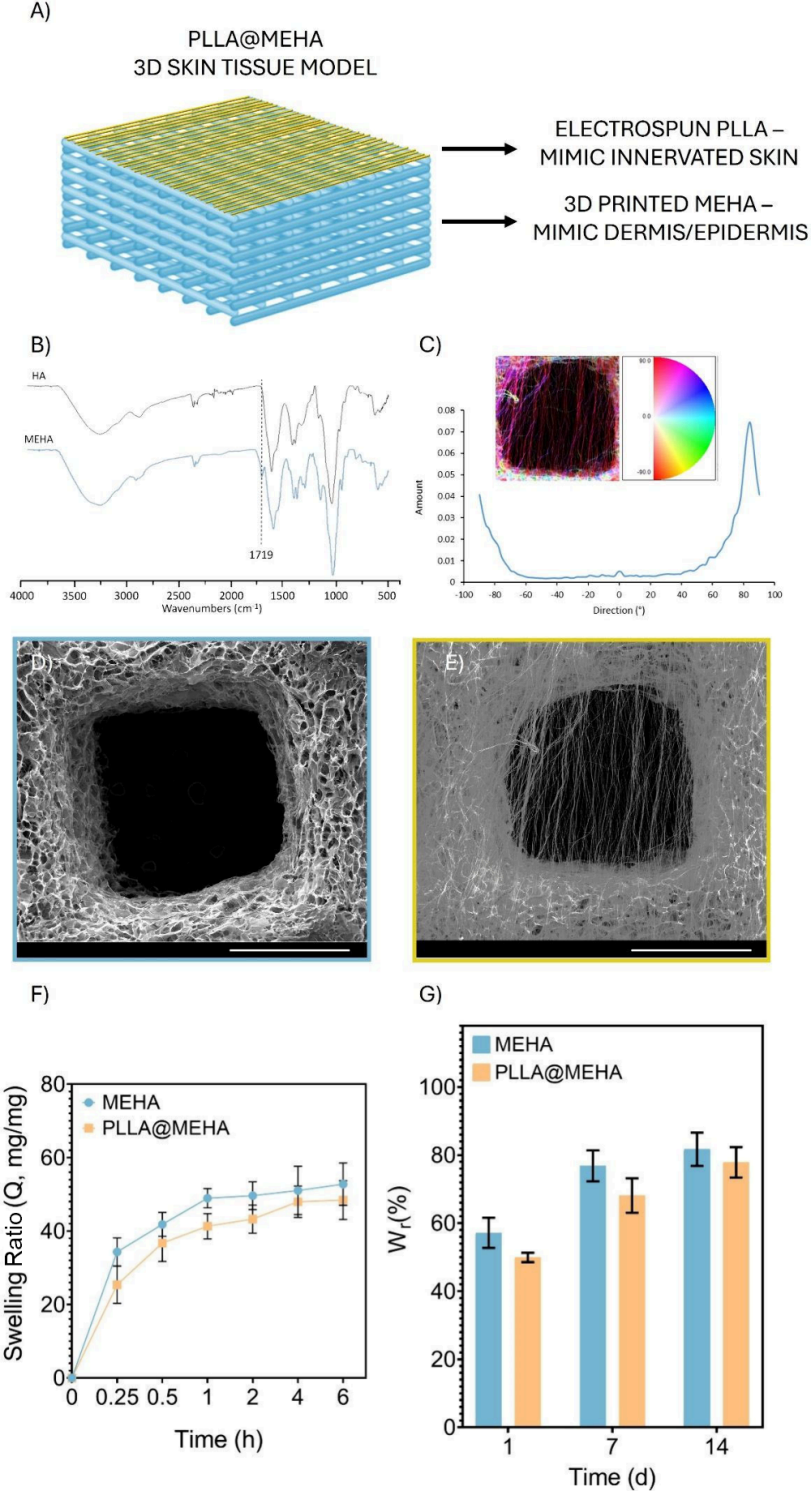
Physicochemical and morphological characterization of a 3D skin model. (A) Representative image of the complex PLLA@MEHA structure. (B) ATR-FTIR spectra of HA and MEHA. (C) Distribution of fibers orientation, with angular values respect to the principal direction. SEM image of (D) MEHA and (E) PLLA@MEHA. Scale Bars: 1 mm. Magnification: 100×. (F) MEHA and PLLA@MEHA swelling ratio in terms of *Q* (mg/mg ± S.E.M.) conducted in antibiotic-supplied phenol red-free DMEM up to 6 h at pH 7.4 and T = 37 °C (n = 5). (G) MEHA and PLLA@MEHA weight loss up to 14 days (% ± S.E.M.) at the same conditions (n = 5).

#### 3.1.1 3D Printing of Methacrylated Hyaluronic Acid (MEHA)

HA is the perfect candidate as a biomaterial for skin tissue engineering applications.^20^ Furthermore, methacrylation has been already widely employed to produce photo-cross-linkable HA with enhanced mechanical stiffness, prolonged stability, and slower degradation, without compromising cytocompatibility. Specifically, this chemical modification enabled UV cross-linking during the bioprinting process, allowing for precise shape retention of the printed constructs. Prompted by those positive results, we decided to employ this 3D system as a skin tissue model.

MEHA was first successfully synthesized according to previous works.^30^ ATR-FTIR analysis confirmed the introduction of methacrylic moieties (Figure 1B). Indeed, the spectrum displayed a band between 950 and 1200 cm^–1^, which can be attributed to C–O stretching vibrations (νC–OH). The strong cluster of bands observed in the 1500–1700 cm^–1^ region corresponds to a superposition of amide I and II signals, along with various carbonyl and carboxyl νC=O vibrations. Notably, the band at 1719 cm^–1^ is characteristic of the νC=O stretch associated with the methacrylic moiety.^31^ Subsequently, MEHA structures were fabricated by 3D printing, a widely adopted, precise, and customizable technology capable of processing a variety of biomaterials.^32^ This approach enabled the creation of a porous 3D well-defined structure with complex and controllable macro- and micro-architectures (Figure 1D).

#### 3.1.2 Electrospinning of Polylactic Acid (PLLA)

Electrospun films exhibit high porosity, facilitating efficient oxygen and nutrient exchange. Moreover, their fibrous architecture closely mimics ECM, which enhances cell adhesion and proliferation.^25^ In the present work, fibers exhibited a homogeneous cylindrical shape, without the presence of beads or other morphological defects and with an average diameter of 0.58 ± 0.09 μm. Moreover, fibers showed parallel organization and were mainly distributed at an angle of 80°. The number of fibers within a fixed distance orthogonal to the fiber alignment direction (#/μm) was 0.11 (Figure 1C,E).

#### 3.1.3 Swelling/Stability and Mechanical Behavior of PLLA@MEHA Model

Swelling and stability represent key properties when designing *in vitro* models. Indeed, the ability to rehydrate in cell culture medium and resemble the perfect milieu for cells is of pivotal importance. Furthermore, the materials should be resistant enough to the handling during the *in vitro* study.^33^

Our 3D complex model reached the equilibrium state in the first hour with a value of *Q* of 48.9 (MEHA) and 41.3 (PLLA@MEHA) without significant differences, thus gaining a mass roughly 40 times greater than its original dry weight (Figure 1F), in agreement with previous works.^20^ The PLLA@MEHA skin model highlighted a similar trend, but slightly lower values of *Q* (41.3) were detected as result of the hydrophobic PLLA, that in turn controlled the swelling behavior. In addition, the 3D printed structure showed an extended degradation time up to 14 days (Figure 1G), without showing signs of delamination. The findings indicated that the PLLA@MEHA was suitable for midterm applications since they swelled significantly in culture media while maintaining their 3D structure over time. Furthermore, by selecting PLLA, which possesses good mechanical and hydrophobic characteristics, as the polymer for electrospinning, the resulting structures exhibited enhanced structural integrity and improved functionality. Therefore, the use of microfibers, due to their remarkable intrinsic properties and designed morphology, can act both as reinforcements and as guidance facilitating the formation of highly aligned cellular constructs.^34^

The mechanical behavior of the 3D complex model was investigated by a uniaxial compression test to evaluate its capacity to withstand externally applied forces while preserving structural integrity. Mechanical stability is a fundamental requirement for engineered skin constructs, as they must tolerate deformation during handling and interaction with surrounding tissues while simultaneously providing an appropriate biomechanical microenvironment for resident cells.

Representative stress–strain curves for MEHA and PLLA@MEHA are shown in Figure S1. At low strain values, a well-defined linear elastic region is observed, corresponding to a mechanical response that is initially stiff. This region is followed by a decrease in stiffness after which a second stiffening phase appears at higher strain levels. Such a three-stage mechanical behavior has been widely reported for different 3D structures fabricated by fused deposition modeling.^20–22,35–37^ The 3D complex model incorporating the PLLA electrospun membrane (PLLA@MEHA) exhibited a compression modulus of 34.3 ± 4.2 kPa, which was higher than that of MEHA alone (28.1 ± 1.5 kPa). Similarly, the maximum compressive stress sustained by PLLA@MEHA (42.7 ± 4.4 kPa) exceeded that of MEHA (35.5 ± 4.7 kPa). These results clearly demonstrate the reinforcing effect of the PLLA electrospun membrane, which enhances the overall mechanical stability of the composite construct without compromising its compliant nature.

Importantly, the mechanical performance of the engineered construct was intentionally designed to approximate that of native human skin. Human dermal tissue is characterized by relatively soft mechanical behavior, with reported Young’s modulus values typically ranging from 10 to 50 kPa.^20,21,36,37^ This mechanical range is critical for regulating key cellular processes, including adhesion, migration, proliferation, and differentiation, which are highly sensitive to the substrate stiffness. By achieving mechanical properties within this physiologically relevant range, the PLLA@MEHA construct effectively reproduced the compliant biomechanical environment of the native dermal tissue.

Overall, this balance between enhanced mechanical robustness and skin-like compliance is essential for the development of a biomimetic skin model. The improved mechanical stability provided by the PLLA reinforcement ensured structural integrity during manipulation, while the preserved softness supported the appropriate cellular behavior. Consequently, this mechanical compatibility was expected to improve both the biological performance and the translational relevance of the engineered skin model for *in vitro* studies and prospective therapeutic applications

### 3.2 Biological Characterization of 3D Skin Model

#### 3.2.1 Cell Growth and Morphology in 2D Conditions

To realize 3D artificial skin, cell lines were previously characterized under 2D culture conditions. Results on cell growth and morphology of fibroblasts isolated from skin biopsies showed ALS fibroblast proliferation rate was significantly reduced (about 50%) compared to HDF proliferation rate, after 7 days of cell culture (#*p* ≤ 0.0001 vs control) (Figure 2A). Meanwhile, SH-SY5Y cells showed the same behavior of HDF cells. Qualitative analysis performed through the fluorescence technique shows morphological features of each cell line (Figure 2B). Specifically, HDF cells showed the expected normal spindle-shaped morphology after 7 days of cell culture (Figure 2B­(a–c)); meanwhile, Slow ALS fibroblasts displayed enlarged nucleoli (Figure 2B­(d–f)), as a result of inflamed conditions. Finally, Fast ALS fibroblasts showed not only enlarged nucleoli but also an increased cell body size and a nontapered shape (Figure 2B­(g–i)). Differentiated SH-SY5Y cells showed the characteristic neuron-like morphology (Figure 2B­(j–l)). Additionally, GAP-43 expression as a marker of neuronal differentiation was quantified on SH-SY5Y cells treated with RA to test SH-SY5Y maturation before their seeding on the PLLA layer of 3D skin constructs. Results reported in Figure S2A revealed that compared to controls (cells without RA), RA treatment increased the expression of GAP-43, a hallmark of neuron maturation, in SH-SY5Y cells. Furthermore, from morphological point of view, SH-SY5Y cells differentiated with RA showed a clear neuron-like morphology, while undifferentiated controls remained flat, polygonal, and epithelial-like with only very short processes (Figure S2B).

**Figure 2:**
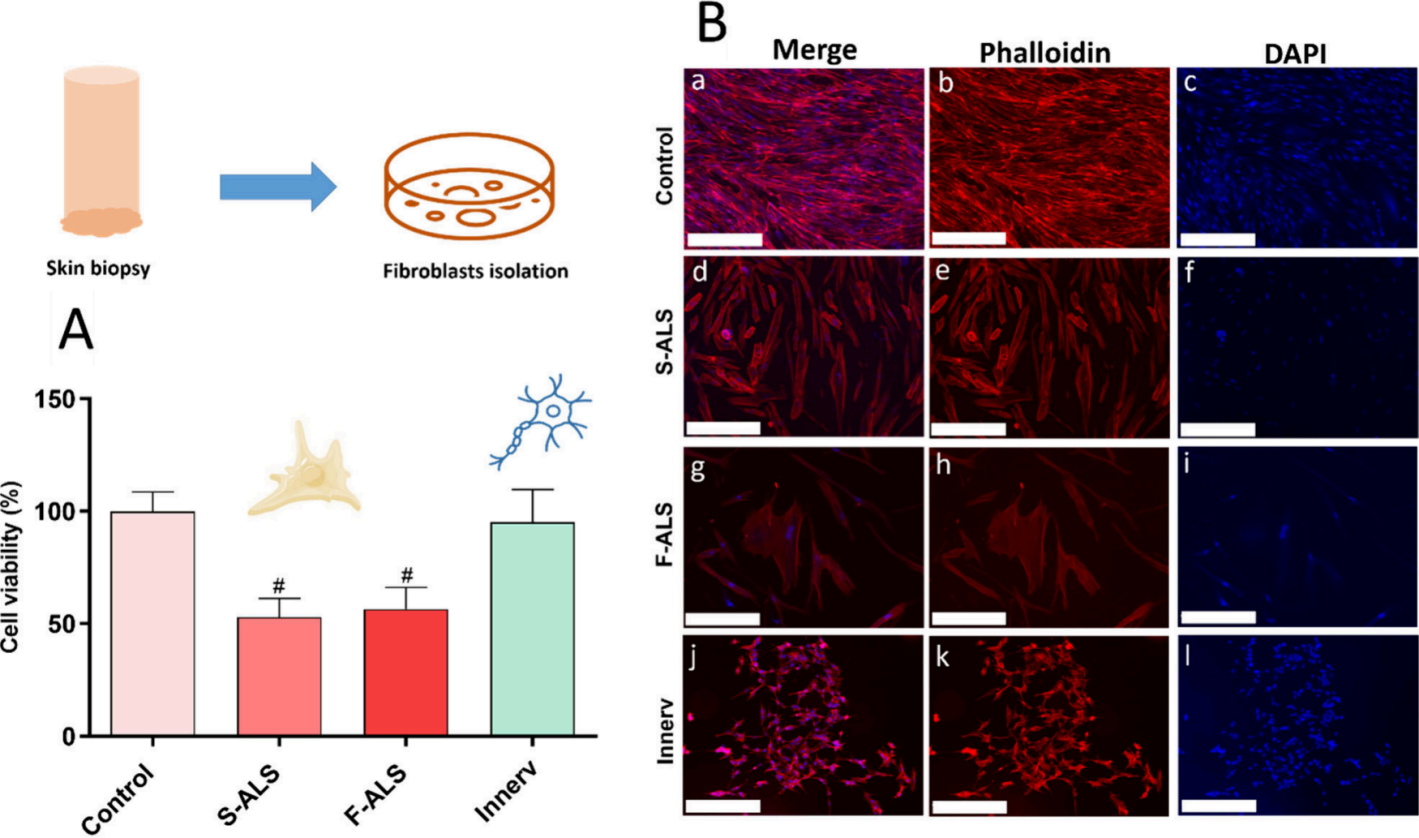
Cell growth and morphology of cell lines used in a 3D skin model. (A) Viability of fibroblasts isolated from skin biopsies and SH-SY5Y in 2D conditions. ALS fibroblast growth was significantly (^#^ *p* ≤ 0.0001 vs control) reduced compared to the HDF one after 7 days of cell culture; SH-SY5Y cells showed the same growth of HDF cells. (B) Morphological analysis of HDF cells (a = merged; b = cytoskeleton; c = nuclei), Slow ALS fibroblasts (S-ALS; d = merged; e = cytoskeleton; *f* = nuclei), Fast ALS fibroblasts (F-ALS; g = merged; h = cytoskeleton; i = nuclei), and SH-SY5Y cells (Innerv; j = merged; k = cytoskeleton; l = nuclei).

#### 3.2.2 Fibroblast Marker Expression in 2D Conditions

Dermal fibroblasts were characterized in terms of specific markers’ expression by fluorescence analysis. Figure 3A shows the expression of Vimentin (green), a specific fibroblast cytoskeletal component, in fibroblasts isolated from Slow and Fast ALS biopsies compared to HDF, after 7 days of cell culture (Figure 3A). Similarly, we reported in Figure 3B, the Hsp47 signal, usually expressed in skin fibroblasts, playing a crucial role in collagen biosynthesis. A significant increase of Hsp47 signal was observed in both Slow and Fast ALS, as reported in image analysis (Figure 3C), indicating the formation of connective-fibrous tissue without function, composed of a mass of collagen fibers. Vimentin and Hsp47 expression, measured using fluorescence analysis, confirmed fibroblasts phenotype of cells isolated from the skin biopsies. Furthermore, our analyses, performed on isolated fibroblasts and compared to HDF, supported a previous study from Riancho et al.^38^ In this work, the authors demonstrated that ALS-fibroblasts exhibited a decreased proliferation rate compared to controls and a high susceptibility to DNA damage.^38^ To analyze the close connection between ALS neurodegenerative mechanisms and neuroinflammatory processes linked to the activation of inflammasome cascade, p-TDP-43 and NLRP-3 expression were quantified in skin isolated fibroblasts using Western blot analysis. Figure S3A shows that p-TDP-43 gradually increases by increasing ALS disease severity (*p ≤ 0.05 and ****p ≤ 0.0001 vs control; #*p* ≤ 0.0001 vs Slow ALS). At the same time, an increase in NLRP-3 expression (Figure S3B) was also observed with significant results in fibroblasts derived from Fast ALS patients (*p ≤ 0.05 vs control). All these results together with the differences in morphology and the abnormal accumulation of specific markers such as p-TDP-43, a pathological hallmark of ALS, suggest ALS derived fibroblasts mirror relevant cellular features of ALS neurodegeneration and neuroinflammation processes, thus making dermal fibroblasts a relevant and accessible ALS cellular model for investigating pathogenetic pathways from a molecular perspective.

**Figure 3:**
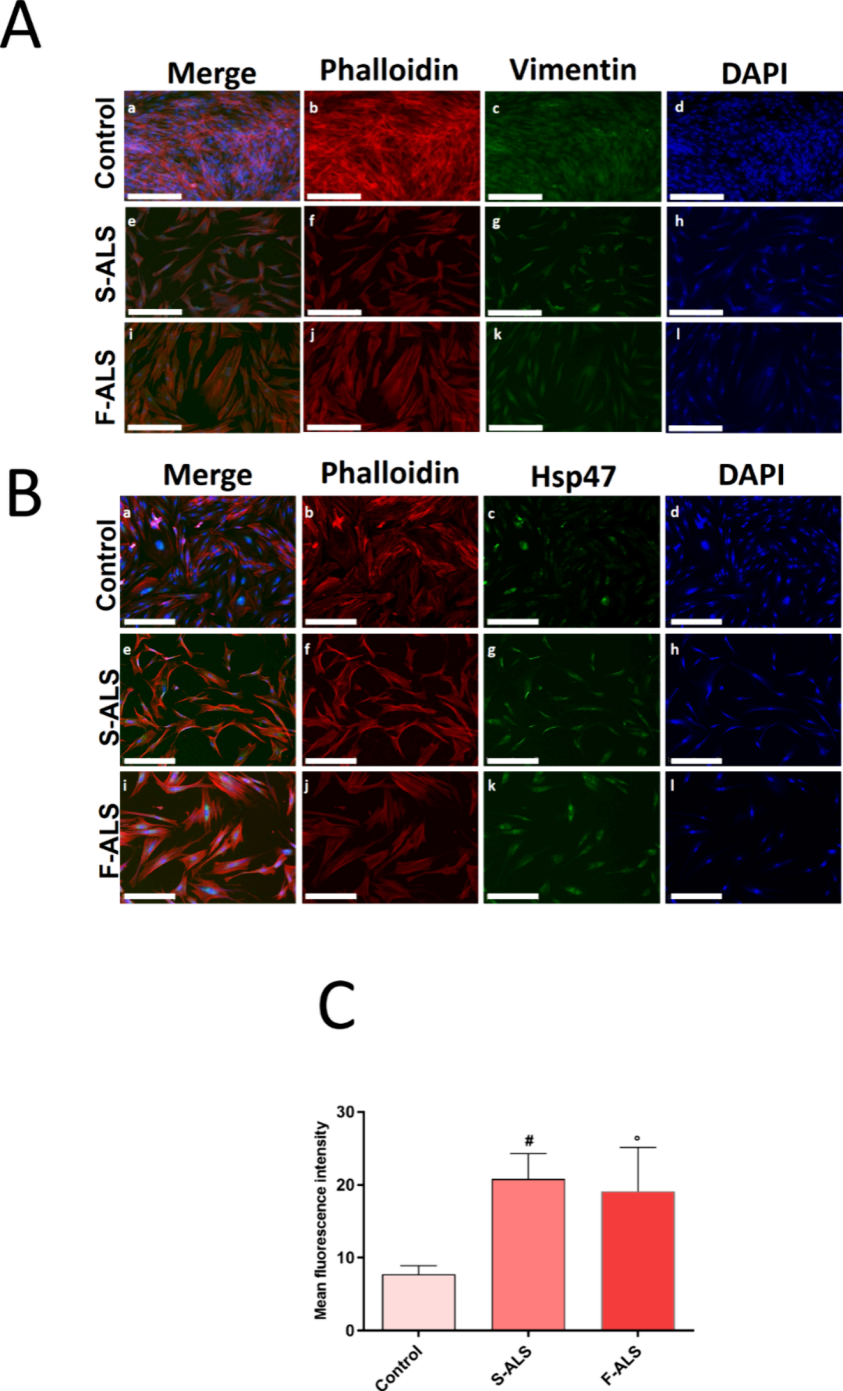
Expression of fibroblast-specific markers in skin biopsy-derived cells. Following fixation, specific antibodies were used to stain the cells. (A) Anti-Vimentin (in green) and anti-Phalloidin-ATTO 594 (F-actin, in red) staining. Nuclei were detected by DAPI (in blue) staining (a,e,i = merged; b,f,j = cytoskeleton; c,g,k = vimentin; d,h,l = nuclei). (B) Anti-Hsp47 (in green) and anti-Phalloidin-ATTO 594 (F-actin, in red) staining. Nuclei were detected by DAPI (in blue) staining (a,e,i = merged; b,f,j = cytoskeleton; c,g,k = Hsp47; d,h,l = nuclei). Magnification: 10×. Scale bar: 250 μm. (C) Image analysis of Hsp-47 expression (^#^ *p* ≤ 0.0001 and °*p* ≤ 0.001 vs control). Fluorescence intensity was normalized to the number of cells per unit area. Data are presented as the mean ± S.D., and the images shown are representative of three independent experiments.

#### 3.2.3 3D Skin Tissue Biocompatibility

Biocompatibility of the 3D artificial skin model was evaluated in terms of cell viability and morphology. Results on cell viability showed that the 3D model promoted each cell line survival after 7 days of culture. Specifically, cell growth maintained the trend observed in 2D conditions with lower values for Slow and Fast fibroblasts than HDF controls (Figure 4A). However, the 3D system allows to appreciate significant differences also between Slow and Fast ALS fibroblasts (Figure 4A). Figure 4B shows morphological features of each fibroblast cell line (HDF, Slow, and Fast ALS fibroblasts) at the interface with neuronal cells that mimic the innervation. The images suggest the 3D skin model is functional in reproducing innervated skin, and it allows the interaction between the several cell phenotypes which constitute *in vivo* skin tissue. SEM analysis (Figure 4C) performed on MEHA surface confirmed differences in fibroblast cell morphology observed in 2D conditions. Specifically, HDF stored the expected normal spindle-shaped morphology after 7 days of cell culture (Figure 4B­(d)), meanwhile Slow and Fast ALS fibroblasts displayed enlarged bodies (Figure 4B­(e, f)), as a result of inflamed conditions.

**Figure 4:**
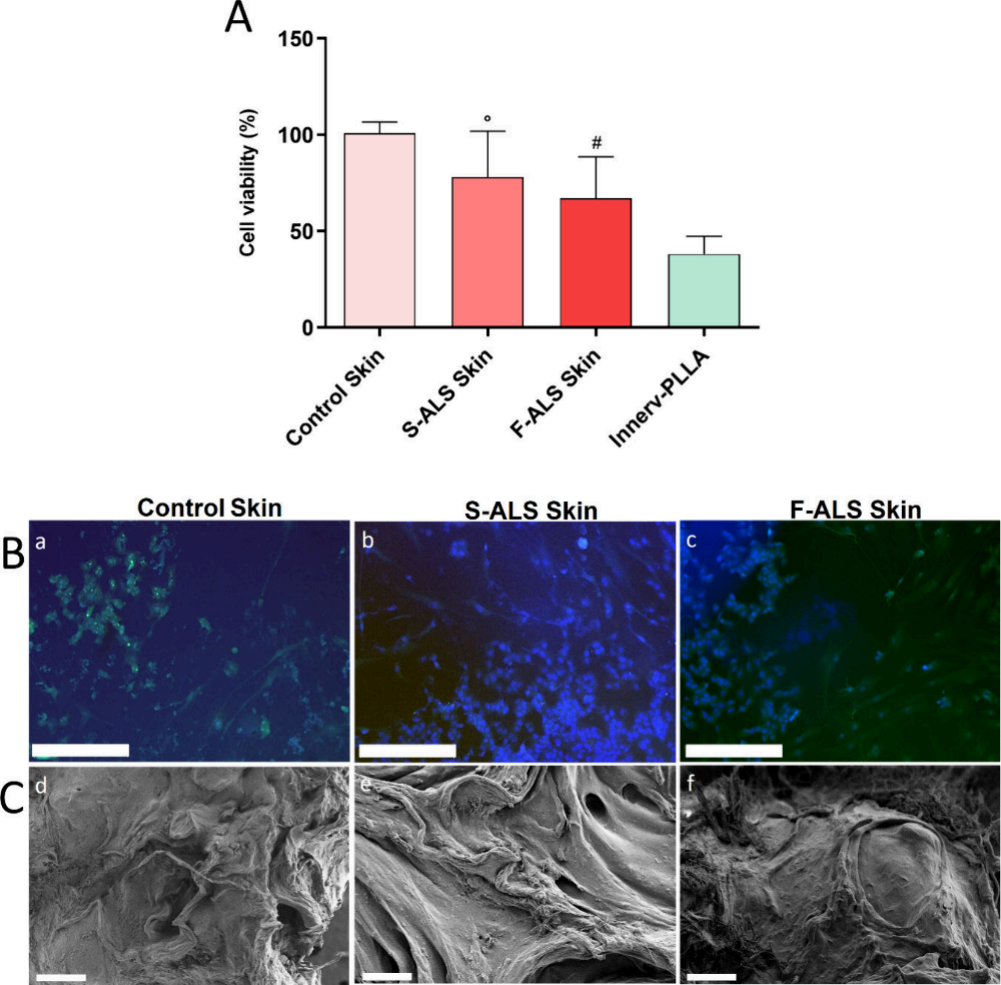
Cell viability and morphology onto 3D artificial skin model. (A) Cell viability of HDF controls (control skin) and Slow (S-ALS Skin) and Fast (F-ALS Skin) fibroblasts cocultured with SH-SY5Y (Innerv-PLLA) onto 3D artificial skin models for 7 days (°*p* ≤ 0.001 and ^#^ *p* ≤ 0.0001 vs control). (B) Morphological features of each fibroblast cell line [HDF (a), Slow (b), and Fast (c) ALS fibroblasts] at the interface with neuronal (SH-SY5Y) cells. Results are mean ± S.D. and the images are representative of three independent experiments. Magnification: 10×. Scale bar: 250 μm. (C) SEM validation of differences in fibroblast cell morphology [HDF (d), Slow (e), and Fast (f) ALS fibroblasts]. Scale bar: 100 μm.

#### 3.2.4 Evaluation of Inflammasome Marker (NLRP-3) Related to Neurodegeneration (p-TDP-43)

During the past few decades, excitotoxicity, oxidative stress, mitochondrial dysfunction, and aggregation of misfolded proteins have been identified as possible potential ALS biomarkers, belonging to different molecular pathways involved in motor neuron degeneration. Indeed, there is considerable evidence to support the involvement of neuroinflammation (activated microglia, astrogliosis, and immune cell infiltration) in ALS abnormalities and progression. Recent results in ALS disease models demonstrate that the activation of the inflammasome cascade and subsequent pyroptosis induction play key roles in cell death and neurodegeneration. However, there is no evidence of this phenomenon on human experimental models, only on mouse models. Herein, we provide the first experimental data derived from human models, demonstrating the involvement of the pyroptosis induction in ALS neurodegeneration. In the literature, only one study has reported on the expression and distribution of inflammasome components and pyroptosis effector proteins in *post-mortem* brain and spinal cord tissues from ALS patients (n = 25) and controls (n = 19).^100^ Previous studies on mouse models hypothesized that NLRP-3 inflammasome activation could induce nerve degeneration in ALS. Therefore, its component levels could predict the disease progression.^11,12^

To better understand the pathology in ALS patients from a molecular perspective and monitor its progression for an efficient treatment, our primary objective in this work was to thoroughly analyze the inflammasome pathway involvement and activation in skin tissue. The aberrant aggregation of TDP-43 in neurons and glia is the defining pathological hallmark of ALS. Nowadays, the structures of pathological (phosphorylated isoform) p-TDP-43 aggregates have been detected only *post-mortem* in the frontal and motor cortices, as well as in the frontal cortex of two distinct ALS patients.^39^ In the present work, for the first time, we have assessed the presence of pathologically aggregated TDP-43 protein on a 3D skin model obtained using fibroblasts isolated from skin biopsies of alive ALS patients. Indeed, actually, 3D models of brain, spinal cord, and muscle derived from cells obtained from ALS patients [often via induced pluripotent stem cells (iPSCs)] have been developed to study neurodegeneration and neuromuscular connection.^40^ However, standard 3D skin models (epidermal/dermo-epidermal equivalents, skin bioprinted constructs) have been developed only for toxicological, cosmetic, and regenerative purposes or for dermatological diseases and skin cancers but not as “ALS-specific 3D skin”.

Here, fluorescence and image analyses revealed an increase of p-TDP-43 (Figure 5A,B) and NLRP-3 (Figure 6A,B) in artificial skin of ALS patients, thus suggesting pyroptosis activation related to a neurodegenerative marker. Specifically, p-TDP-43 is a marker of ALS aggressiveness because its expression increases by increasing ALS severity. Particularly, the findings in this study revealed that NLRP-3, as components of the inflammasome cascade, may be involved in ALS pathogenesis and its gene expression levels could be used as a biomarker for better predicting ALS onset and progression not only in mouse models but also in ALS patients.

**Figure 5:**
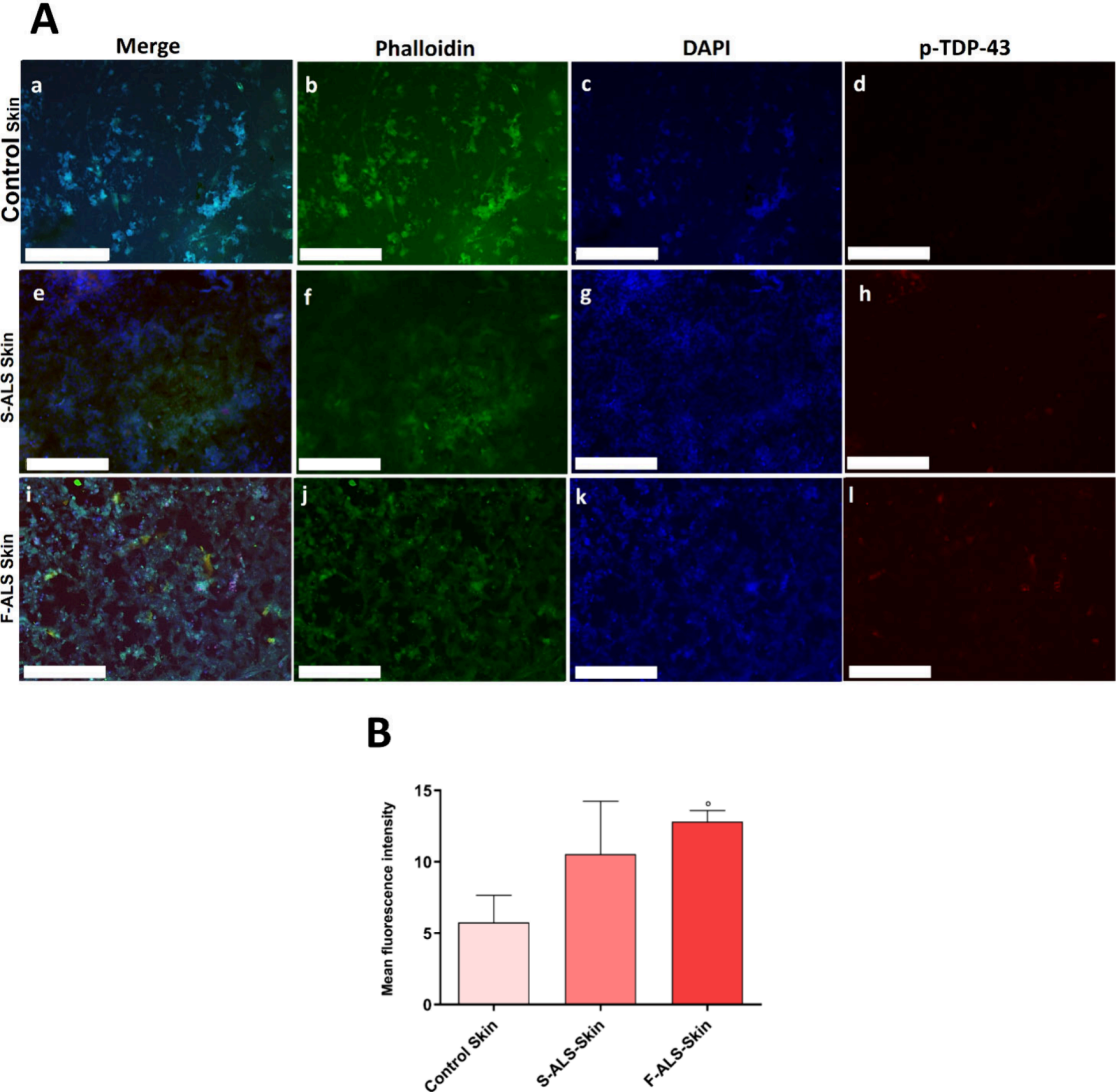
p-TDP-43 neurodegenerative marker expression in 3D skin model. Following fixation, specific antibodies were used to stain the cells. (A) Anti-p-TDP-43 (in red) and anti-Phalloidin-FITC (F-actin, in green) staining. Nuclei were detected by DAPI (in blue) staining (a,e,i = merged; b,f,j = cytoskeleton; c,g,k = nuclei; d,h,l = p-TDP-43). Magnification: 10×. Scale bar: 250 μm. (B) Image analysis of p-TDP-43 expression (°*p* ≤ 0.001 vs control). Fluorescence intensity was normalized to the number of cells per unit area. Data are presented as mean ± S.D., and the images shown are representative of three independent experiments.

**Figure 6:**
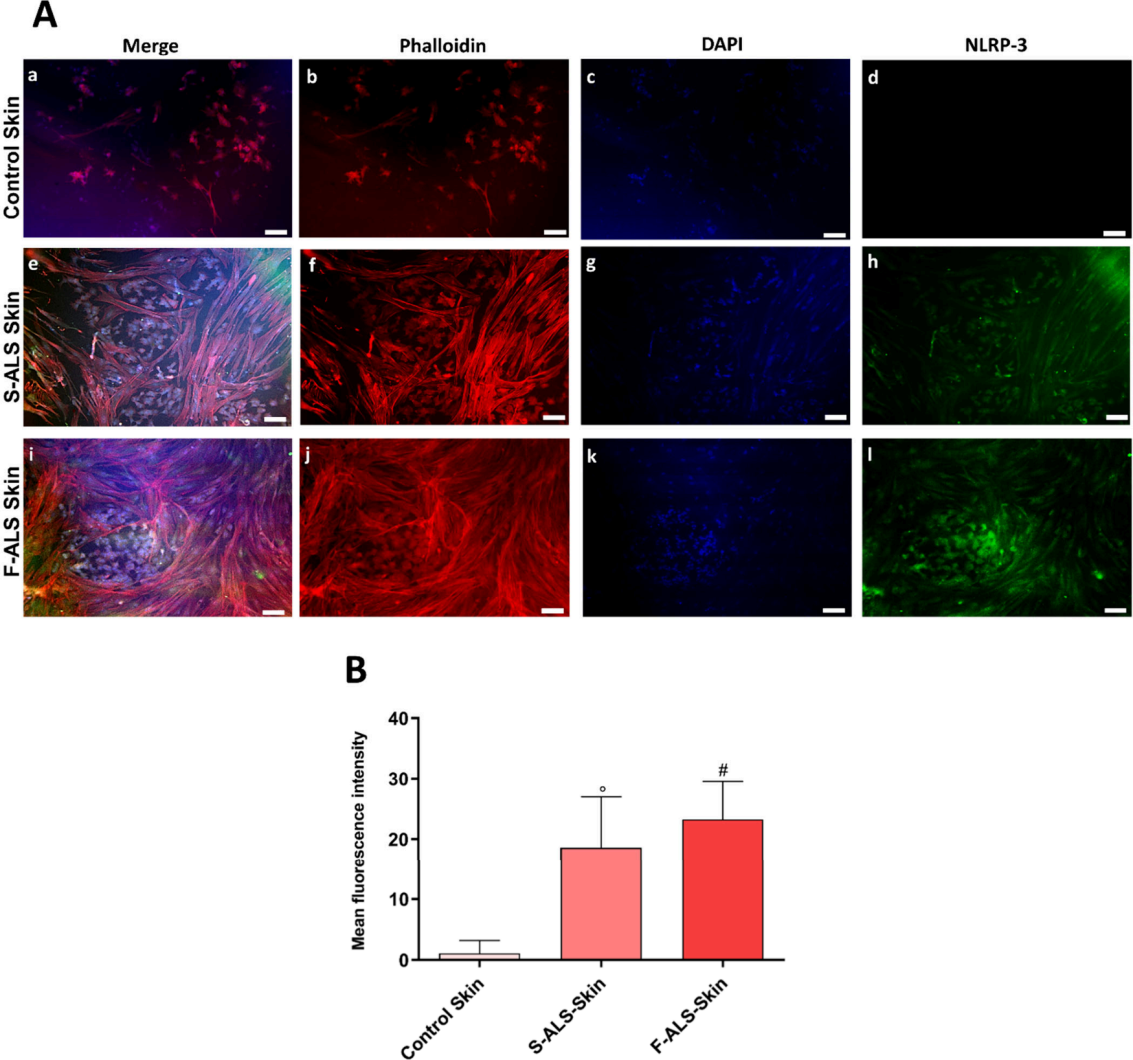
NLRP-3 as inflammasome marker expression in 3D skin model. Following fixation, specific antibodies were used to stain the cells. (A) Anti-NLRP-3 (in green) and anti-Phalloidin-ATTO 594 (F-actin, in red) staining. Nuclei were detected by DAPI (in blue) staining (a,e,i = merged; b,f,j = cytoskeleton; c,g,k = nuclei; d,h,l = NLRP-3). Magnification: 10×. Scale bar: 100 μm. (B) Image analysis of NLRP-3 expression (°*p* ≤ 0.001 and ^#^ *p* ≤ 0.0001 vs control). Fluorescence intensity was normalized to the number of cells per unit area. Data are presented as mean ± S.D., and the images shown are representative of three independent experiments.

#### 3.2.5 Cytokines Production Related to NLRP-3 Inflammasome Activation on ISF

An upregulated inflammatory response in neurodegenerative disorders causes the release of proinflammatory cytokines and metabolites of nitric oxide (NO), which play a pivotal role for triggering neuroinflammation and controlling redox balance, which results in neurodegeneration.^41^ Furthermore, NLRP-3 inflammasome is a multiprotein complex that, upon activation by a large range of stimuli, activates Caspase-1 that in turn mediates the maturation of the pro-inflammatory cytokines including IL-6 and IL-18.^13^ Among all cytokines, IL-18 appears to be the most relevant mediator of inflammasome-driven cascade activation in ALS patients.^42^ Indeed, inflammasome is important for inducing elevation of IL-18 plasma levels in a murine model.^43^ Our findings proved that artificial ISF, consisting of factors released in culture medium by Fast and Slow ALS fibroblasts colonizing the artificial skin tissue, allowed us to understand that nitrites (Figure 7A) and IL-6 (Figure 7B) increased with increasing ALS aggressiveness. Furthermore, both Slow and Fast ALS progressors released higher IL-18 than controls (Figure 7C), suggesting IL-18 as a disease-relevant inflammatory marker (°*p* ≤ 0.001 and ^#^ *p* ≤ 0.0001 vs control; *p ≤ 0.05 and ^+^ *p* ≤ 0.0001 vs Slow ALS). These results were compared to those obtained in 2D conditions, underlining that the 3D skin model allowed us to appreciate more significant differences between fibroblasts of ALS patients and their controls. Indeed, concerning nitrites levels, no differences between Slow and Fast ALS were detected (an increased nitrites production compared to control, ^#^ *p* ≤ 0.0001 vs control), as reported in Figure 7D. By contrast, in 3D skin model nitrites levels increased by increasing ALS severity (Figure 7A). Regarding IL-6 that is a generic proinflammatory marker also involved in pyroptosis, no differences were observed between 2D and 3D results (^#^ *p* ≤ 0.0001 vs control and ^+^ *p* ≤ 0.0001 vs Slow ALS). Finally, Figure 7F showed no differences in IL‑18 levels between the control and Slow ALS; significant IL-18 production was detected only in FastALS (°*p* ≤ 0.001 vs control and ^#^ *p* ≤ 0.0001 vs Slow ALS). Instead, results on this cytokine production obtained in 3D skin model revealed higher levels of IL-18 in the ISF. Specifically, both Slow and Fast ALS progressors released about 600 pg mL^–1^ of IL-18 compared to healthy control (about 60 pg mL^–1^). This result was corroborated by IL-18 quantification performed on the overall cohort consisting of three Slow progressors and three Fast progressors (Table S1 and Figure S4). This latter result confirmed the importance of IL-18 as a relevant biomarker of inflammasome activation in ALS.

**Figure 7:**
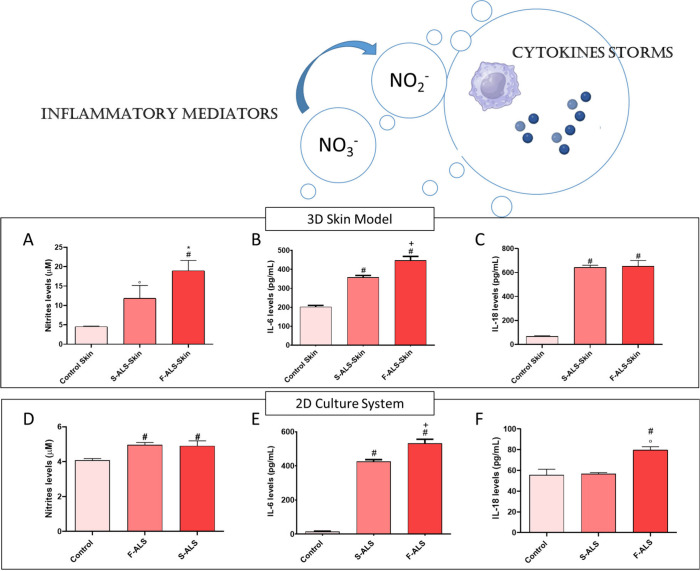
Nitrite and cytokine production after the inflammasome cascade activation. (A) Nitrite, (B) IL-6, and (C) IL-18 levels in 3D skin model (°*p* ≤ 0.001 and ^#^ *p* ≤ 0.0001 vs control; *p ≤ 0.05 and ^+^ *p* ≤ 0.0001 vs Slow ALS). (D) Nitrite, (E) IL-6, and (F) IL-18 levels in 2D conditions (°*p* ≤ 0.001 and ^#^ *p* ≤ 0.0001 vs control; ^+^ *p* ≤ 0.0001 and ^#^ *p* ≤ 0.0001 vs Slow ALS). Results are mean ± S.D. of 3–4 experiments.

## 4 Conclusions

In this study, we developed a patient-derived 3D innervated skin model that provides a physiologically relevant human platform to investigate ALS pathophysiology under standardized conditions while reducing the need for repeated biopsies. The model successfully reproduced key disease features, including the activation of the NLRP-3 inflammasome pathway and accumulation of p-TDP-43, both of which increased with disease severity. Importantly, the engineered tissue produced interstitial fluids enriched in pyroptosis-related mediators. Among these, IL-18 levels were consistently elevated in both Slow and Fast progressors compared with controls, supporting its potential role as a disease-related inflammatory biomarker. In parallel, the progressive increase in p-TDP-43 confirmed its association with neurodegenerative burden and clinical aggressiveness. Compared with conventional 2D cultures, the 3D system enabled a more sensitive discrimination of disease profiles and better reproduced *in* *vivo*-like cellular interactions. It is worth noting that in the present manuscript, ALS-mimicking patients were not considered, but the comparison between 3D skin models derived from ALS and ALS-mimicking patients will be part of a future study. However, the overall findings highlight the value of this platform for biomarker discovery, disease stratification, and preclinical therapeutic testing, providing a proof of concept that engineered human skin models can serve as translational tools for ALS research. In the near future, this approach may support the development of cutaneous biosensor strategies aimed at monitoring disease progression in a minimally invasive manner, reducing diagnostic delay, and enabling more personalized patient management.

## Supplementary Material



## Data Availability

The authors declare that all data supporting the findings of this study are available within the paper; source data for the figures in this study are available from the authors upon request.

## Notes

Mechanical behavior of 3D printed PLLA@MEHA model, GAP-43 expression as marker of differentiated SH-SY5Y cells, skin biopsy-derived cells express p-TDP-43 and NLRP-3, demographic and clinical characteristics of patients, additional IL-18 levels in Slow and Fast progressors (PDF)
