# Supplementary material for: 3D Artificial Skin Model As a Novel Strategy for the Detection of Pyroptosis-Cascade Activation in Amyotrophic Lateral Sclerosis
Source: ACS Appl Mater Interfaces. 2026 Mar 6;18(10):14603–16. doi: 10.1021/acsami.5c23366 (PMC13006939; doi:10.1021/acsami.5c23366)
Supplement: Supplementary file 1 [file am5c23366_si_001.pdf]

Supporting Information

3D Artificial Skin Model as a Novel Strategy for the Detection of Pyroptosis-cascade Activation in Amyotrophic Lateral Sclerosis

Enrico Scarpa[1#], Ugo D'Amora[1*#], Noemi De Cesare[1], Irene Bonadies[2], Raffaele Dubbioso[3], Maria Nolano[4], Principia Dardano[5], Luca De Stefano[5], Alessandra Fasolino[6], Stefania Zeppetelli[1], Alessandro Silvestri[7], Chiara Zanardi[7], Evelina Milella[1], Ines Fasolino[1*]

[1] Institute of Polymers, Composites and Biomaterials – National Research Council (IPCB-CNR), Viale Kennedy 54, Mostra d'Oltremare pad. 20, 80125, Naples, Italy

[2] Institute of Polymers, Composites and Biomaterials – National Research Council (IPCB – CNR), Via Campi Flegrei 34, 80078 Pozzuoli, Italy

[3] Department of Neurosciences, Reproductive Sciences and Odontostomatology, University of Naples Federico II, Via Sergio Pansini, 5, 80131 Naples, Italy

[4] Laboratorio Biopsie di Cute, Istituti Clinici Scientifici Maugeri Spa SB - IRCCS Telese Terme, Via Bagni Vecchi, 1, 82037 Telese Terme (BN), Italy

[5] Institute of Applied Sciences and Intelligent Systems – National Research Council (ISASI – CNR), Via Pietro Castellino, 111, 80131, Naples, Italy

[6] Clinical Neurophysiology Unit "A. Cardarelli Hospital", 9, 80131, Naples, Italy

[7] Ca' Foscari University of Venice, Department of Molecular Sciences and Nanosystems, via Torino 155, 30172 Venezia, Italy

* Corresponding author: ines.fasolino@cnr.it

Co-corresponding: ugo.damora@cnr.it

#Equally contributed

Table S1. Demographic and clinical characteristics of patients.

**Variable**	**Patient 1**	**Patient 2**	**Patient 3**	**Patient 4**	**Patient 5**	**Patient 6**
Clinical profile	S-ALS	S-ALS	S-ALS	F-ALS	F-ALS	F-ALS
Sex	F	F	M	F	F	M
Age	67	50	75	78	67	69
Phenotype	Flail leg	Flail leg	Flail arm	Bulbar	Classical	Pyramidal
FVC (%)	92	89	53	75	NA	29
NIV	No	No	No	No	Yes	Yes
PEG	No	No	No	No	No	Yes
Disease duration (months)	18	7	83	7	27	25
Rate of progression	0.44	0.4	0.13	1.71	1.04	1.64
Diagnostic delay (months)	15	7	48	7	20	11
BMI	23.14	20.75	26.89	18.67	22.31	23.78
ALSFRS-R	40	45	37	36	20	7
King B	1	0	0	1	1	1
King U	0	0	1	1	1	1
King L	1	1	1	1	1	1
King total	2	1	2	3	4B	4A-B

Abbreviations: FVC, forced vital capacity. BMI, body mass index. NIV, non invasive ventilation. PEG, percutaneous endoscopic gastrostomy. ALSFRS-R, Amyotrophic Lateral Sclerosis Functional Rating Scale

Revised, NA, not assessed. King B, bulbar involvement score in the King staging system. King U, upper limb involvement score in the King staging system. King L, lower limb involvement score in the King staging system. King total, overall King clinical stage derived from the number of anatomical regions involved and the presence of nutritional or respiratory failure. Stages range from 1 to 4, with stage 4 subdivided into 4A for nutritional failure requiring PEG and 4B for respiratory failure requiring ventilatory support. Rate of progression was calculated using the standard ALS progression index as follows: Rate of progression = (48 – ALSFRS R score at evaluation) divided by disease duration in months since symptom onset. This index reflects the monthly functional decline, with higher values indicating faster disease progression. The slow profile (S-ALS) was defined for patients in King's stage 1 or 2 with a rate of progression below 0.5, whereas the fast profile (F-ALS) profile was defined for patients in stage 3 or 4 with a rate of progression above 1.

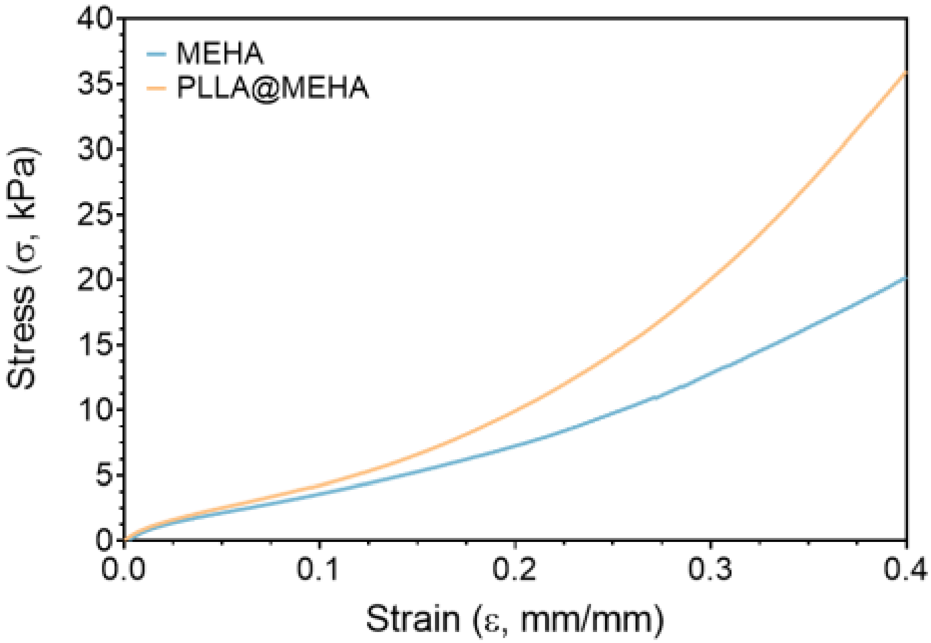

Figure S1. Mechanical behavior of 3D skin model.
Typical stress-strain curves obtained from compression tests on MEHA and PLLA@MEHA models (rate of 1 mm×min^{-1}), final strain of 40%.

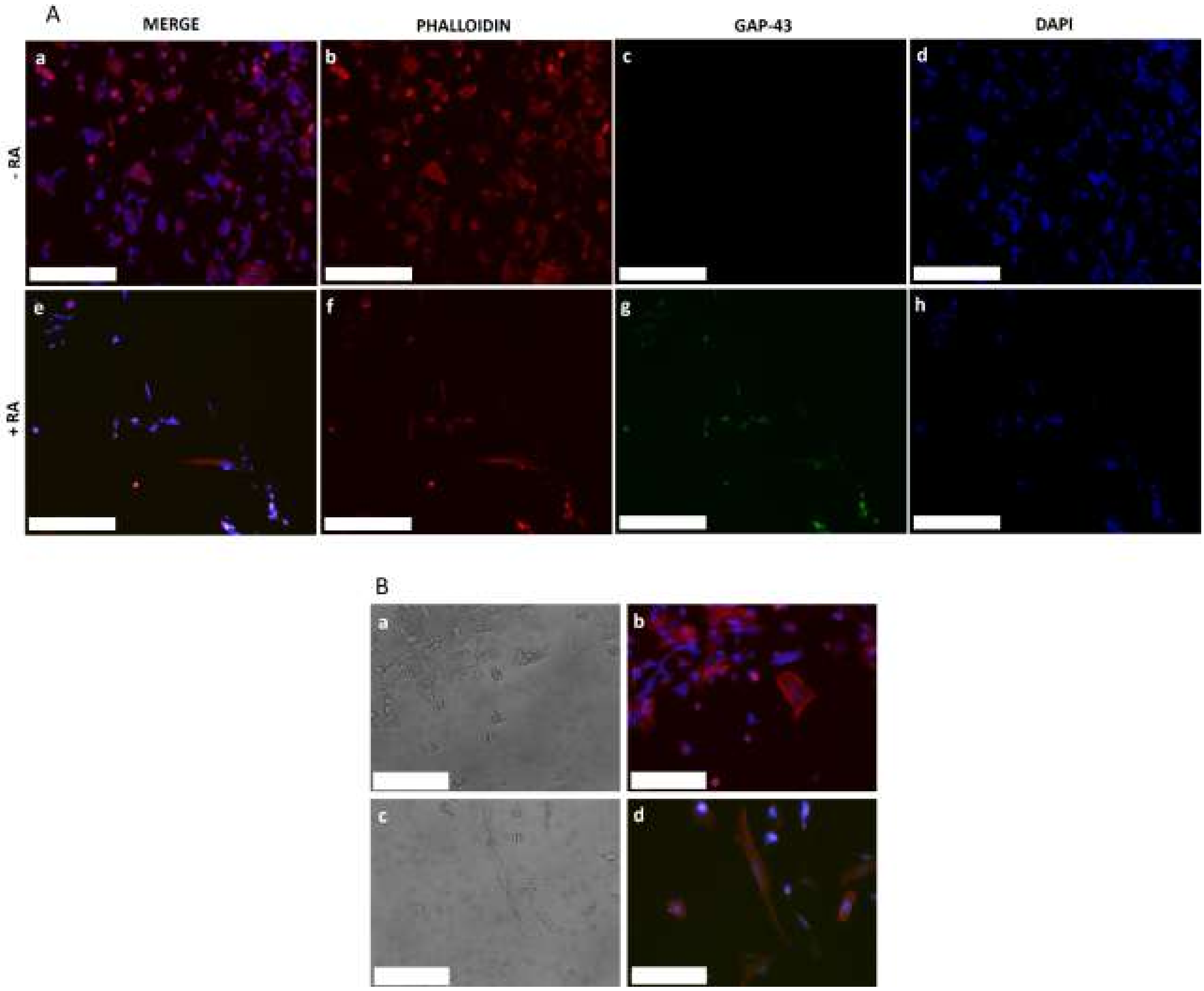

Figure S2. GAP-43 expression as marker of mature neurons derived from pre-neuronal SH-SY5Y cells after treatment with retinoic acid (RA).

(A) Fluorescence analysis on cell differentiation was performed using the GAP-43 marker expression. Upon fixation, cells were stained with specific antibodies: Anti-GAP-43 (in green) and anti- Phalloidin-ATTO 594 (F-actin, in red) staining; nuclei were detected by DAPI (in blue) staining (a,e = merged; b,f= cytoskeleton; c,g,= GAP-43; d,h = nuclei. Magnification 10× Scale bar: 125 µm. (B) Morphological features of SH-SY5Y cells without (a,b) and with (c,d) RA treatment using optical (a,c) and fluorescence (b,d) analysis. Magnification 20× Scale bar: 125 µm.

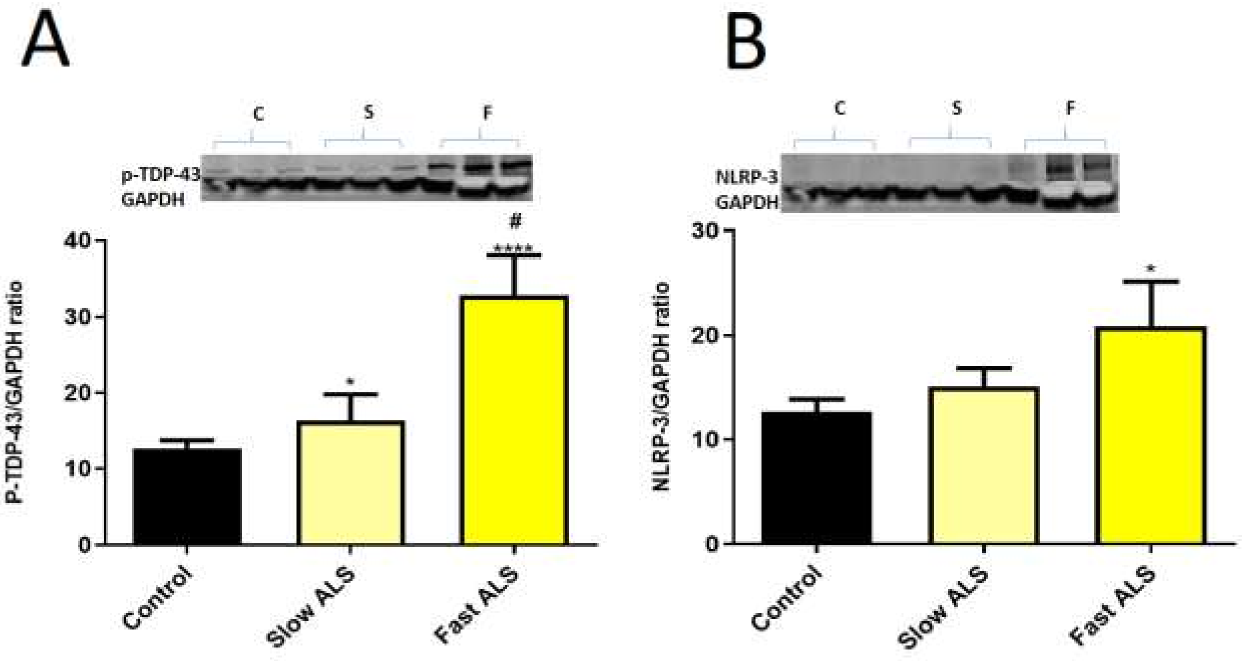

Figure S3. Skin biopsy–derived cells express p-TDP-43 and NLRP-3.
(A) p-TDP-43 and (B) NLRP-3 through western blot analysis on skin isolated fibroblasts and their controls. C, S and F stand for Control, Slow and Fast progressors. Results are mean ± S.D. of 3-4 experiments, *p≤0.05 and ****p≤0.0001 vs Control; #p≤0.0001 vs Slow ALS.

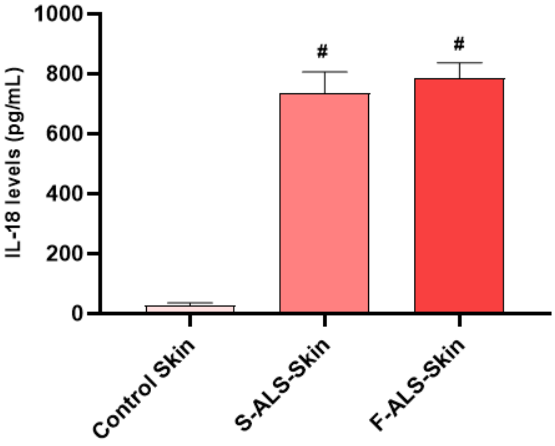

Figure S4. IL-18 ELISA validation.
IL-18 levels of the overall cohort reported in Table S1 ($^{\#}p \leq 0.0001$ vs control). Results are mean ± S.D. of 3 experiments.
